# Radiation recall dermatitis: case report and review of the literature

**DOI:** 10.3747/co.2008.201

**Published:** 2008-01

**Authors:** A.E. Hird, J. Wilson, S. Symons, E. Sinclair, M. Davis, E. Chow

**Affiliations:** * Rapid Response Radiotherapy Program, Department of Radiation Oncology, Toronto–Sunnybrook Regional Cancer Centre, Toronto, Ontario; † Medical Oncology, Humber River Regional Hospital, Toronto, Ontario; ‡ Department of Radiology, Toronto–Sunnybrook Regional Cancer Centre, Toronto, Ontario

**Keywords:** Radiation recall dermatitis, breast cancer, orbital metastases

## Abstract

“Radiation recall”—also called “radiation recall dermatitis”—has been defined as the “recalling” by skin of previous radiation exposure in response to the administration of certain response-inducing drugs. Although the phenomenon is relatively well known in the medical world, an exact cause has not been documented. Here, we report a rare occurrence of the radiation recall phenomenon in a breast cancer patient after palliative radiotherapy for bone, brain, and orbital metastases.

## 1. HISTORY

A 55-year-old woman was diagnosed with breast adenocarcinoma in August 2006. In late September, she complained of back pain and slight numbness. A bone scan revealed a mild increase in activity in the thoracic spine and the proximal fourth and anterior sixth ribs. Magnetic resonance imaging (mri) of the spine confirmed metastatic involvement in the tenth thoracic vertebra. The patient received radiotherapy with 20 Gy in 5 fractions in October 2006. She tolerated the treatment very well with complete pain relief.

**FIGURE 1:**
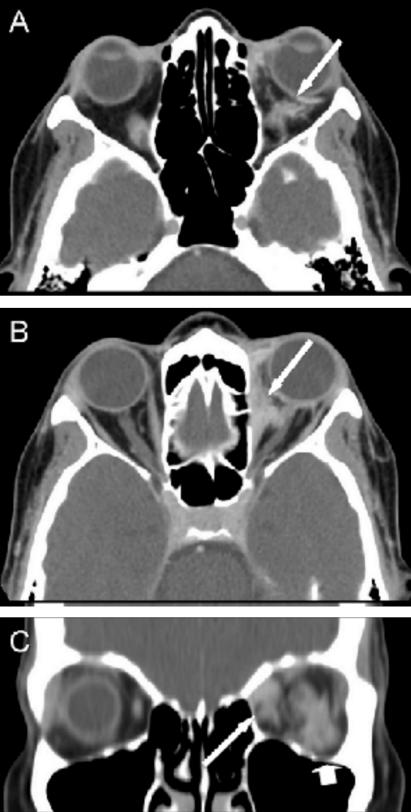
Retrobulbar metastasis, axial, and coronal computed tomography images with contrast. (A) Axial view of the inferior orbits demonstrates enhancing abnormal soft tissue posterior and lateral to the left globe (arrow). (B) Axial view of the upper orbits demonstrates enhancing abnormal soft tissue in the medial left orbit inseparable from the medial rectus muscle (arrow). (C) Coronal view demonstrates abnormal soft tissue in the medial left orbit inseparable from the medial rectus (arrow) and in the lateral inferior left orbit inseparable from the inferior rectus and in contact with the optic nerve (arrowhead).

The woman returned to her medical oncologist for systemic therapy. A combination of paclitaxel (175 mg/m^2^) and gemcitabine (1000 mg/m^2^) was commenced in November 2006. After receiving a single dose, the patient complained of double vision. A computed tomography examination confirmed left orbital metastases (Figure 1) and multiple intraparenchymal brain metastases in the left frontal lobe and left cerebellum. The woman was treated with whole-brain radiotherapy (wbrt), including the left orbit, which received a dose of 20 Gy in 5 fractions. The initial chemotherapy treatment took place 13 days before the commencement of the wbrt. No adverse reactions were observed immediately after the radiation treatment.

Ten days after completion of the wbrt, the patient received her second dose of paclitaxel and gemcitabine. Within 2 days, the patient detected discoloured and inflamed skin limited to the region that had previously been irradiated. She also experienced swelling in the left ear, muffled hearing, and discomfort in the eyes as a result of the reaction. Surprisingly, increased pigmentation also occurred in the area of the thoracic bone metastases treated with palliative radiotherapy approximately 7 weeks earlier. Silver sulphadiazine cream and hydrocortisone eardrops were prescribed to treat external symptoms. All chemotherapy was put on hold.

Approximately 4 weeks after development of the skin reaction, the patient developed new cervical nodes compatible with clinical progression of her breast cancer. Once the external skin reaction had improved significantly, with only mild discolouration remaining, chemotherapy was resumed. At this time, nearly 6 weeks had passed since the appearance of the radiation recall dermatitis (rrd). A chemotherapy regimen of cyclophosphamide (600 mg/m^2^), epirubicin (100 mg/m^2^), and 5-fluorouracil (600 mg/m^2^) replaced the paclitaxel and gemcitabine. Dexamethasone (Decadron: Merck, Whitehouse Station, NJ, U.S.A) was administered at 20 mg before the first chemotherapy treatment and at 10 mg before each subsequent treatment. No adverse reactions have occurred since. At follow-up, the patient’s double vision had improved, and a computed tomography scan revealed a stable appearance in the orbital metastases. New mri examination of the brain, orbits, and spine revealed no demyelination corresponding to the areas affected by the rrd reaction.

## 2. DISCUSSION

“Radiation recall”—also called rrd—is defined as the “recalling” by skin of previous radiation exposure in response to the administration of certain response-inducing drugs 1. In the medical world, the rrd phenomenon has been termed anything from “moderately rare” to “moderately common.” No exact cause or incidence has been documented^2^.

D’Angio and colleagues originally documented rrd in 1959 3; the trigger for the abnormal reaction was dactinomycin 1. Cytotoxics are common instigators 1. Some medications have been documented to be more commonly involved with rrd: docetaxel, doxorubicin, gemcitabine, and paclitaxel (Tables I and II). Although the association is only a loose one, Camidge and Price proposed that more-severe skin reactions are more common when the period between radiation and the recall-triggering drug is smaller 1. According to Putnik *et al.* 60, the median time between the conclusion of radiation treatment and the materialization of rrd is 39 days. In the present case, materialization of the rrd occurred within 2 days.

Although the precise mechanism of action for rrd is not known, several mechanisms that may, or may not, lead to the development of radiation recall have been proposed. These mechanisms include changes in vascularization, dna repair, radiation-impaired epithelial function of stem cells, and increased sensitivity to drugs 1. Corticosteroids have been suggested to have some protective effects 61. We found that steroids are commonly used in the treatment of external symptoms and with the intention of preventing recurrent reactions during subsequent chemotherapy administration 23,25,26,30,32,33,39,40,43,45,46,50,53,55,59,60.

Most recall reactions have occurred when radiotherapy and chemotherapy are separated by less than 2 months (Table I). The present case demonstrates a maximum time frame of 7 weeks separating radiation and resumption of chemotherapy treatment. Of the total reported cases of rrd outlined here, only 27% (20/75) demonstrated a duration greater than 7 weeks in terms of time passed between completion of radiotherapy and commencement of chemotherapy 1,5–7,12,13,17,19,20,23,24,27,30,37,39,52,53,59.

Although rrd is a rare phenomenon, it poses a significant barrier to treatment for patients. The condition creates a paradox: patients and clinicians alike wish to proceed with the most desirable treatment in the given circumstances, but are unable to do so because of the rare skin reaction. The present report serves as a reminder to palliative health care professionals of the possible danger of a recall reaction if an insufficient period has passed between radiotherapy and commencement of a potential recall-inducing drug.

## Figures and Tables

**TABLE I:** I Radiation recall dermatitis (rrd): case summaries

Reference	Condition treated	Radiation dose	Drug leading to rrd^a^	Time to rrd	Treatment a
Tan *et al.*, 1959 4	Ewing sarcoma of the left hip	10 Gy to the left knee and 17.5 Gy to the spine	Dactinomycin (75 μg/kg) 7 days after completion of rt	Unspecified	Unspecified
D’Angio, 1962 3	Wilms tumour	rt to the left lung and right paracardiac	Dactinomycin	During administra- tion of response- inducing drug	Unspecified
Von Essen *et al.*, 1963 5	Breast carcinoma	30 Gy	5-Fluorouracil (15 mg/kg daily for 4 days) 7 weeks after rt	2 Weeks	Unspecified
Sears, 1964 6	Wilms tumour	Postsurgical tumour-bed irradiation	Hydroxyurea (60 mg/kg daily) 1 month after rt	5 Days	Unspecified
	Wilms tumour	Radiation for pulmonary metastasis	Hydroxyurea (60 mg/kg daily) 1 month after rt	9 Days	Unspecified
	Rhabdomyosarcoma of the cervical area	30 Gy	Hydroxyurea (60 mg/kg daily) 47 days after rt	8 Days	Unspecified
	Rhabdomyosarcoma of the cervical area	16 Gy to site of pulmonary metastasis	Hydroxyurea (60 mg/kg daily) 7 days after rt	16 Days	Unspecified
Lampkin, 1969 7	Rhabdomyosarcoma of the right middle ear	56.44 Gy to the right face 28.32 Gy to the left side	Vinblastine (0.2 mg/kg) 2 months after rt	1 Day	Unspecified; same reaction occurred 2 weeks later
Jaffe *et al.*, 1973 8	Osteogenic sarcoma	26.25 Gy	Methotrexate (400 mg/kg) 24 hours after rt	Unspecified	Unspecified; rechallenged 2–3 weeks later with no recurrence
Donaldson *et al.*, 1974 9	Fibrosarcoma of the right mandible	59.5 Gy	Doxorubicin (Adriamycin: 60 mg/m^2^) 5 weeks after rt	7 Days	Unspecified; rechallenged at weeks 7 and 15 at the same and reduced doses with identical reaction
	Osteosarcoma of the fibula	59.5 Gy	Doxorubicin (Adriamycin: 60 mg/m^2^) 1 week after rt	7 Days	Unspecified; rechallenged after 12 weeks with identical reaction
Etcubanas and Wilbur, 1974 10	Mandibular fibrosarcoma	Unspecified	Doxorubicin (Adriamycin: 60 mg/m^2^) 4 weeks after rt	Unspecified	Unspecified; after second cycle, a milder skin reaction occurred
	Unspecified	Unspecified	Doxorubicin (Adriamycin: 60 mg/m^2^) 1 week after rt	Unspecified	Unspecified; rechallenged twice, with identical reaction each time
Cassady *et al.*, 1975 11	Lymphoma to the right axilla and supraclavicular fossa	12 Gy to mantle field	Doxorubicin (Adriamycin: 75 mg/m^2^) 26 days after rt	Hours	Unspecified
	Osteosarcoma to the left proximal humerus	24 Gy	Doxorubicin (Adriamycin: 30 mg/m^2^ daily for 3 days) 18 days after rt	7 Days	Unspecified
Rosen *et al.*, 1975 12	Osteogenic sarcoma	16 Gy	Methotrexate (200 mg/kg) 8 weeks after rt	5 Days	Unspecified; rechallenged twice with similar reactions
Mayer *et al.*, 1976 13	Metastatic breast cancer	45 Gy to the spine	Doxorubicin (Adriamycin: 80 mg/injection, 1 injection per month for 9 months) given 7 years after rt	7 Months (at the time of the 7th injection)	Unspecified
Fontana, 1979 14	Small-cell lung cancer	38 Gy	Etoposide (100 mg/m^2^ on days 1–3) 7 days after rt	18 Hours	Unspecified; rechallenged 3 weeks later, resulting in the same reaction
Solberg *et al.*, 1980 15	Acute myelomonocytic leukemia and leukemia cutis	21 Gy whole-body irradiation	Doxorubicin (Adriamycin: 35 mg/m^2^ daily for 3 days) given 2 days after rt	4 Days	Death related to toxicity
Weiss *et al.*, 1986 16	Advanced cancers	Unspecified	Intravenous trimetrexate (80 mg/m^2^ over 24 hours) every 28 days	Unspecified	Unspecified
		Unspecified	Intravenous trimetrexate (200 mg/m^2^ over 24 hours) every 28 days	Unspecified	Unspecified
		Unspecified	Intravenous trimetrexate (200 mg/m^2^ over 24 hours) every 28 days	Unspecified	Unspecified
		Unspecified	Intravenous trimetrexate (200 mg/m^2^ over 24 hours) every 28 days	Unspecified	Unspecified
Jolivet *et al.*, 1987 17	Lung cancer	40 Gy	Trimetrexate (2 mg/m^2^ bolus) for 9 consecutive days every 28 days for 2 cycles; begun 10 months after completion of rt	Unspecified	Unspecified
Kellie *et al.*, 1987 18	Embryonal rhabdomyosarcoma of the legs	54 Gy	Melphalan (200 mg/m^2^) 6 weeks after completion of rt	24 Hours	Unspecified
Nemechek and Corder, 1992^19^	Man (age 34) with hiv and a large Kaposi sarcoma lesion on the left foot	27 Gy in 15 fractions	Intravenous vinblastine (10 mg/m^2^), begun 10 months after rt	48 Hours	Healed by the 5th day after chemotherapy
Parry, 1992 20	Woman (age 70) with breast cancer	Wide local excision and adjuvant rt 2 years earlier	Tamoxifen (20 mg daily) started 2 years after rt	5 Days	Discontinued tamoxifen; resolved in 2 weeks; rechallenged at 10 mg daily, with mild recurrence
Raghavan *et al.*, 1993 21	Recurrent breast carcinoma	61.2 Gy to the chest wall; 65.3 Gy to the supraclavicular region	Paclitaxel (130 mg/m^2^ over 24 h), begun 2 days after rt	5 Days	Antibiotics, chest wall debridement
Stelzer *et al.*, 1993^22^	aids-related Kaposi sarcoma	Each lesion randomized to 1 of 3 possible radiation fractionation schemes:1) 40 Gy in 20 fractions2) 40 Gy in 20 fractions3) 8 Gy in 1 fraction4) 20 Gy in 5 fractions	Intravenous bleomycin (10 mg/m^2^) on a weekly basis	1) 3 Days after second injection2) 3 Days after second injection3) No rrd4) No rrd	In 1) and 2), exacerbated by oral etoposide therapy started 4 days after appearance of the skin reaction
Shenkier and Gelman, 1994, as cited by	Advanced gastric cancer	44 Gy	Paclitaxel (90 mg/m^2^) 7 months after rt	3 Hours	Mild recurrence when paclitaxel given in 7 further cycles
Camidge and Price, 2001 1	Advanced gastric cancer	44 Gy	Paclitaxel (90 mg/m^2^) 8 months after rt	6 Hours	No recurrence when paclitaxel given in 7 further cycles
Abadir and Liebmalen 1995 23	Woman (age 60) with adenocarcinoma of the gallbladder	Tumour dose was 61.2 Gy in 34 fractions	Simvastatin for hypercholesterolemia (20 mg daily), 11 months after rt	2–3 Days	Prednisone and cephalotin
Extermann *et al.*, 1995 24	Man (age 55) with ductal carcinoma of the right breast	Tamoxifen (20 mg daily), plus 48.25 Gy with a 15-Gy boost to the tumour bed	Isoniazid 400 mg, plus rifampicin 600 mg, plus pyrazinamide 2 mg to treat nasopharyngeal tuberculosis, 4 months after rt	During 4th week of treatment	All medications were continued, and the reaction gradually regressed in the weeks following
Perez *et al.*, 1995 25	Woman (age 32) with metastatic breast cancer	30 Gy to the lumbar spine	Edatrexate (100 mg/m^2^ biweekly), begun 6 weeks after rt	After 3 doses (11 days)	Topical therapy, nsaids; rechallenged with prednisone with mild recurrence
Phillips, 1995, as cited by Camidge and Price, 2001 1	Unknown	25 Gy	Paclitaxel (90 mg/m^2^) given 27 days after rt	3 Days	No recurrence when paclitaxel given in 3 further cycles
Schweitzer *et al.*, 1995 26	Woman (age 61) with metastatic lung adenocarcinoma	43.2 Gy to the mediastinum; 46 Gy to the ribs	Paclitaxel (175 mg/m^2^ over 3 hours), begun 12 days after rt completion	Hours	Dexamethasone (Decadron: 20 mg), diphenhydramine (50 mg); paclitaxel given with Decadron after 2 weeks with no recurrence
Bokenmeyer *et al.*, 1996 27	Woman (age 55) with breast cancer	50 Gy to the breast; 54 Gy to the lymph nodes	Paclitaxel (175 mg/m^2^ over 3 h), 13 months after rt	5 Days	Discontinuation of paclitaxel
McCarty *et al.*, 1996 28	Woman (age 51) with invasive lobular breast carcinoma	50.4 Gy in 28 fractions; mild skin erythema developed	Paclitaxel (200 mg/m^2^), 7 days after rt	4 Days	Healing within 10 days; treatment unspecified
Yeo *et al.*, 1997 29	Woman (age 51) with breast cancer	30 Gy in 10 fractions to T10–L4 spine and pelvis	Docetaxel (100 mg/m^2^) on 3-weekly basis and prior oral dexamethasone (Decadron) for 5 days	4 Days after second injection	Dose reduction; no recurrence of rrd
Bostrom *et al.*, 1999 30	Woman (age 48) with highly differentiated tuboloductal breast cancer	54 Gy	Tamoxifen (20 mg/m^2^ daily) 28 months after rt	2 Months	Local steroid cream, mometasone furoate, once daily for 10 days; skin appeared normal 7 weeks after discontinuing tamoxifen; after 8 weeks, restarted on toremifene without recurrence
Wilson *et al.*, 1999 31	Woman (age 46) with breast cancer	Unspecified	Epirubicin	2 Weeks	Surgical debridement and microvascular free-flap reconstruction
Camidge & Kunkler, 2000 32	Woman (age 50) with breast cancer	50 Gy in 20 fractions	Cycle 2 of docetaxel (100 mg/m^2^) with dexamethasone (Decadron: 8 mg once daily for 3 days), 16 days after end of rt	Within 7 days	Docetaxel reduced to 75% and given 21 days later; steroids for 7 days without recurrence
Castellano *et al.*, 2000 33	Man (age 61) with stage iv nsclc	24 Gy in 8 fractions	Gemcitabine (1250 mg/m^2^ on days 1, 8, 15 per 28-day cycle) 4 weeks after completion of rt	8 Days (second dose)	Oral dexamethasone (Decadron) and diphenhydramine; resolved 10 days later; treatment continued with other chemotherapies
Giesel *et al.*, 2000 34	Woman with breast cancer	Whole-brain irradiation: 2 Gy for 5 days weekly, up to 50 Gy	Docetaxel restarted (30 mg/m^2^ weekly)	Unspecified	Unspecified
	Woman with breast cancer	Whole-brain irradiation: 2 Gy for 5 days weekly, for up to 50 Gy	Docetaxel re-started (100 mg/m^2^ weekly)	Unspecified	Unspecified
Kharfan *et al.*, 2000 35	Woman (age 25) with stage iv nodular sclerosis Hodgkin disease	30 Gy to lumbar spine and right proximal femur	Methotrexate (10 mg/m^2^ on day +1 after bone marrow transplant; 15 mg/m^2^ on days +3, +6, and +11)	7 Days after transplant	Hydrating emulsions (treated symptomatically)
Chan *et al.*, 2001 36	Man (age 50) with a sigmoid carcinoma	41.4 Gy in 23 days	Oxaliplatin-based chemotherapy (oxaliplatin, plus 5-fluorouracil, plus folinic acid), resumed 8 days after completion on rt	3 Days	Aqueous cream and sodium fusidate ointment (Fucidin); chemotherapy discontinued and reaction settled after 2 weeks; 5-fluorouracil plus folinic acid alone resumed without recurrence
Kennedy and McAleer, 2001 37	Malignant melanoma to the right temple	30 Gy	Dacarbazine (800 mg/m^2^ once every 3 weeks), begun 2 months after rt	10 Days	Unspecified
Bar-Sela *et al.*, 2001 38	Man (age 65) with lung adenocarcinoma	RT to mediastinum and upper lobe	Gemcitabine	Unspecified	Unspecified
Jeter *et al.*, 2002 39	Woman (age 41) with breast cancer	30 Gy in 10 fractions to lumbar spine	Gemcitabine (1000 mg/m^2^ every 2 weeks), plus trastuzumab (Herceptin) weekly for 4 weeks, 5.5 months after rt	2 Weeks	Discontinuation of gemcitabine slowly resolved the skin reaction
	Man (age 79) with nsclc	30 Gy in 10 fractions	Gemcitabine (1000 mg/m^2^) 11 days after rt	10 Days	Supportive care, alginate gel pads, bowel rest
	Woman (age 63) with metastatic adenocarcinoma of unknown primary	30 Gy in 10 fractions, plus 25 Gy in 2 fractions (boost)	Gemcitabine (1000 mg/m^2^), 3.4 months after rt	3 Days	Intravenous steroids for 2 days with minimal response
Morkas *et al.*, 2002 40	Woman (age 39) with infiltrating ductal carcinoma	50.4 Gy in 28 fractions, plus 10 Gy to tumour bed and 2 cm surrounding	Docetaxel (100 mg/m^2^), plus prophylactic dexamethasone (Decadron)	10 Days	Methylprednisone (80 mg twice daily); docetaxel at 75% induced a less severe reaction
Ortmann and Hohenberg, 2002 41	Woman (age 56) with breast cancer	30 Gy in 10 fractions to right hip	Capecitabine (2000 mg twice daily for 14 days)	3 Days after completion of first course	Unspecified
Piroth *et al.*, 2002 42	Woman (age 40) with breast cancer	30.9 Gy upper-body irradiation, plus whole-brain and pelvis	Docetaxel (30 mg/m^2^) started 1 week after rt	14 Days	Discontinuation and anti-inflammatory agents
Thomas and Stea, 2002 43	Woman (age 29) with malignant melanoma of the scalp	Excision, plus biweekly treatments of 6 Gy, totalling 30 Gy	Intravenous interferon alfa-2b (20×10^9^ IU) administered 5 days after completion of rt	6 Days	Occlusive dressings with wound gel; resolved in 7 days
Ee and Yosipovitch, 2003 44	Woman (age 55) with metastatic breast cancer	Photo-recall	Chemotherapy with taxanes	Unspecified	Unspecified
Jimeno *et al.*, 2003 45	Woman (age 53) with stage IV infiltrating ductal carcinoma	30 Gy to left femur	Pegylated liposomal doxorubicin (40 mg/m^2^ on day 1 every 28 days), 4 weeks after completion of rt	12 Days	Topical steroids (betamethasone dipropionate); completely resolved 14 days later
Keung *et al.*, 2003 46	Woman (age 49) with breast cancer	50 Gy in 25 fractions following modified mastectomy	Arsenic trioxide (0.15 mg/kg daily), for 5 days each week for 5 weeks	Day 2 of week 3	Arsenic trioxide discontinued, topical triamcinolone/silver sulfadiazine cream started
Schwartz *et al.*, 2003 47	Woman (age 37) with recurrent ovarian adenocarcinoma	Palliative whole-pelvis rt: 45 Gy in 25 fractions	3 Months later, started on gemcitabine (800 mg/m^2^), every other week; reduced to 600 mg/m^2^ because of severe neutropenia	Unspecified	Ciprofloxacin (250 mg twice daily) with slight improvement; 2nd cycle after 2 weeks produced the same reaction within 24 hours
Muggia, 2004^48^	Woman with breast cancer	rt to supraclavicular, internal mammary, and axillary areas	Doxorubicin with weekly trastuzumab	2–4 Weeks	None; continued liposomal doxorubicin
Singer *et al.*, 2004 49	Woman (age 88) with infiltrating ductal carcinoma	50.4 Gy, plus 10 Gy to tumour bed	Tamoxifen (20 mg daily)	3 Months	None; continued on tamoxifen; completely resolved 3 months later
Borgia *et al.*, 2005 50	Woman (age 63) with infiltrating ductal carcinoma	50 Gy over 5 weeks	Docetaxel (100 mg/m^2^), every 3 weeks started 1 week after rt	4 Days after second course	Oral methylprednisone resulted in 10-day complete remission
Kandemir *et al.*, 2005 51	Woman (age 55) with breast cancer	50 Gy over 5 weeks	Docetaxel (100 mg/m^2^), plus oral dexamethasone (Decadron) for 3 days	11 Days	None; complete resolution after 6 days; continued docetaxel with no recurrence
Marisavljevic *et al.*, 2005 52	Woman (age 32) with stage iib Hodgkin lymphoma	Total dose of 60 Gy	Gemcitabine (1250 mg/m^2^ on days 1, 8, 15), plus oral dexamethasone (Decadron: 8 mg on days 1, 2, 8, 9, 15, 16) more than 2 years after rt	2 Days	Skin reaction faded over 10 days without specific treatment; mild recurrence after each gemcitabine administration
Ash and Videtic, 2006 53	Woman (age 56) with infiltrating ductal carcinoma	50 Gy in 25 fractions, plus additional 10 Gy in 5 fractions to lumpectomy site	Phentermine 1 year after rt	Unspecified	Prednisone 30 mg daily for 2 weeks; minimal discolouration after 4 weeks
Ayoola and Lee, 2006 54	Woman (age 54) with lung squamous cell carcinoma	64.8 Gy to thorax and mediastinum	Cefotetan upon admission to hospital for cholecystitis	3 Days	Cefotetan withdrawn; free of pain in 4 days
Barlesi *et al.*, 2006 55	Woman (age 75) with primary lung adenocarcinoma; treated for breast cancer 27 years earlier	Lumpectomy and adjuvant radiation to the breast 27 years earlier	Pemetrexed (500 mg/m^2^); oral prednisone (40 mg) twice daily the day before, the day of, and the day after chemotherapy	3 Days	Steroids (prednisone 1 mg/kg daily); improvement in 48 hours; resolution at 2 weeks
Fakih, 2006 56	White man (age 52) with pancreatic adenocarcinoma	1.8 Gy daily for 50.4 Gy total	Gemcitabine (1000 mg/m^2^), for 3 weeks every 4-week-cycle	Cycle 5	Withdrawal of gemcitabine resulted in spontaneous resolution
Kaya *et al.*, 2006 57	Woman (age 41) with non-Hodgkin lymphoma	UV radiation	Methotrexate (high dose), plus cytarabine (high dose)	Unspecified	Cold compress; lesions resolved within a week (with hyperpigmentation)
Kundranda and Daw, 2006 58	Woman (age 48) with well-differentiated infiltrating ductal carcinoma	50 Gy with a boost of 14 Gy to the tumour bed	Tamoxifen (20 mg daily)	Within 1 week	Oral cephalexin did not provide relief; tamoxifen discontinued, diphenhydramine given; after 12 weeks, restarted on tamoxifen with mild itchiness, but no recurrence
Mizumoto *et al.*, 2006 59	Woman (age 76) with diffuse large B cell lymphoma of the left neck	36 Gy in 18 fractions to the left neck	Docetaxel (60 mg/m^2^) every 3 weeks 1 year after rt	6 Days	Gargle with a local anesthetic and topical corticosteroids; 80% of docetaxel dose was given 2 weeks later; milder recall phenomenon recurred after 1 week
	Woman (age 60) with breast cancer	50 Gy in 20 fractions	Docetaxel (30 mg/m^2^ weekly) restarted 14 days after rt	Day 6 after second course of chemotherapy	Topical corticosteroids; continued docetaxel therapy for 9 cycles
Putnik *et al.*, 2006 60	Man (age 65) with squamous cell carcinoma of the epiglottis	64.8 Gy	Hypericin	4 Weeks after rt, then again 1 year after rt	Symptoms controlled by steroid cream, but disappeared only when hypericin was discontinued
Hird *et al.*, 2007 (present article)	Woman (age 55) with metastatic breast adenocarcinoma	1) 20 Gy in 5 fractions to the thoracic spine (October 2006) 2) Whole-brain radiation: 20 Gy in 5 fractions (November 2006)	Paclitaxel (175 mg/m^2^) and gemcitabine (1000 mg/m^2^) administered 1.5 weeks after completion of whole-brain radiation	2 Days	Silver sulphadiazine cream and hydrocortisone eardrops; discolouration still apparent after 8 weeks; started on cef with concurrent dexamethasone (Decadron) without recurrence

a Holders of named pharmaceutical trademarks: Adriamycin: Pharmacia, Kalamazoo, MI, U.S.A.; Decadron: Merck and Co., Whitehouse Station, NJ, U.S.A.; Fucidin: Leo Pharma, Ballerup, Denmark; Herceptin: Genentech, San Francisco, CA, U.S.A.

rt = radiotherapy; nsaids = nonsteroidal anti-inflammatory drugs; nsclc = non-small-cell lung cancer; uv = ultraviolet; cef = cyclophospha-mide, epirubicin, 5-fluorouracil.

**TABLE II:** Radiation recall dermatitis–inducing drugs (*n* = 75)^1^,^4^–^61^

Drug	Frequency	
	(n)	(%)
Docetaxel	10	13
Doxorubicin	10	13
Gemcitabine	8	11
Paclitaxel	8	11
Trimetrexate	5	7
Methotrexate	4	5
Hydroxyurea	4	5
Tamoxifen	4	5
Dactinomycin	2	3
Vinblastine	2	3
Others	18	24
